# Recommendations for the surgical treatment of endometriosis—part 1: ovarian endometrioma

**DOI:** 10.1186/s10397-017-1029-x

**Published:** 2017-12-19

**Authors:** Working group of ESGE, ESHRE, and WES, Ertan Saridogan, Christian M. Becker, Anis Feki, Grigoris F. Grimbizis, Lone Hummelshoj, Joerg Keckstein, Michelle Nisolle, Vasilios Tanos, Uwe A. Ulrich, Nathalie Vermeulen, Rudy Leon De Wilde

**Affiliations:** 1 Institute for Women’s Health, University College Hospital, Reproductive Medicine Unit, Elizabeth Garrett Anderson Wing, London, NW1 2BU UK; 2 Nuffield Department of Obstetrics and Gynaecology, University of Oxford, Endometriosis CaRe Centre, John Radcliffe Hospital, Women’s Centre, Oxford, OX3 9DU UK; 3 Department of Obstetrics and Gynecology, HFR Fribourg Hôpital Cantonal, 1708 Fribourg, Switzerland; 4 1st Department of Obstetrics & Gynecology, Medical School, Aristotle University of Thessaloniki, Tsimiski 51 Street, 54623 Thessaloniki, Greece; 5 World Endometriosis Society, London, N1 3JS UK; 6 Landeskrankenanstalten-Betriebsgesellschaft (KABEG) and Landeskrankenhaus Villach, Abteilung für Gynäkologie und Geburtshilfe, 9500 Villach, Austria; 7 Hôpital de la Citadelle, Department of Obstetrics & Gynecology, BE-4000 Liège, Belgium; 8 Department of Ob/Gyn, Aretaeio Hospital, 2024 Nicosia, Cyprus; 9 Department of Obstetrics and Gynecology/Endometriosis Center, Martin-Luther Hospital, 14193 Berlin, Germany; 10 ESHRE, Central Office, Meerstraat 60, BE-1852 Grimbergen, Belgium; 11 Carl von Ossietzky Universitat Oldenburg, University Hospital for Gynecology, DE-26129 Oldenburg, Germany

**Keywords:** Endometriosis, Laparoscopy, Surgery, Endometrioma, Cystectomy, Ablation, Electrocoagulation

## Abstract

**Study question:**

What does this document on the surgical treatment of endometriosis jointly prepared by the European Society for Gynaecological Endoscopy (ESGE), ESHRE, and the World Endometriosis Society (WES) provide?

**Summary answer:**

This document provides recommendations covering technical aspects of different methods of surgery for endometriomas in women of reproductive age.

**What is already known:**

Endometriomas (ovarian endometriotic cysts) are a commonly diagnosed form of endometriosis, owing to the relative ease and accuracy of ultrasound diagnosis. They frequently present a clinical dilemma as to whether and how to treat them when found during imaging or incidentally during surgery. Previously published guidelines have provided recommendations based on the best available evidence, but without technical details on the management of endometriosis.

**Study design, size and duration:**

A working group of ESGE, ESHRE and WES collaborated on writing recommendations on the practical aspects of endometrioma surgery.

**Participants/materials, setting and methods:**

This document focused on endometrioma surgery. Further documents in this series will provide recommendations for surgery of deep and peritoneal endometriosis.

**Main results and the role of chance:**

The document presents general recommendations for surgery of endometrioma and specific recommendations for cystectomy, ablation by laser or by plasma energy, electrocoagulation and a combination of these techniques applied together or with an interval between them.

**Limitations and reasons for caution:**

Owing to the limited evidence available, recommendations are mostly based on clinical expertise.

**Wider implications of the findings:**

These recommendations complement previous guidelines on the management of endometriosis.

**Study funding/competing interests:**

The meetings of the working group were funded by ESGE, ESHRE and WES. CB declares to be a member of the independent data monitoring committee for a clinical study by ObsEva and receiving research grants from Bayer, Roche Diagnostics, MDNA Life Sciences and Volition. ES received honoraria for provision of training to healthcare professionals from Ethicon, Olympus and Gedeon Richter. The other authors declare that they have no conflict of interest.

## What does this mean for patients?

This paper was produced by a European working group looking at the different types of surgery for endometriosis, a common condition where tissue, which is similar to the lining of the womb, is found elsewhere in the body.

The working group looked specifically at how best to treat a type of ovarian cyst called an endometrioma, which can occur in women who have endometriosis. Drug therapies may be used to treat endometriosis, but when endometriomas are found and need treatment, surgery is often used. There are risks associated with surgery as it can damage the follicles in the ovaries and reduce fertility.

The working group looked at the main types of surgery which are used to treat endometriomas in women who may want to have children in the future. They considered cystectomy, where the cyst is cut out, ablation, where the cyst lining is removed by using a laser beam or plasma energy, and electrosurgery, where an electric current is used. They also looked at the effectiveness of combining different types of surgery.

The paper discusses in detail how different types of surgery should be performed taking potential risks into consideration and stresses that careful planning is essential to ensure the best outcomes.

## Introduction

Endometriosis is a common inflammatory condition affecting women, mostly during their reproductive years [3]. Endometriosis can be asymptomatic, but associated symptoms include abdominal pain, painful periods, dyspareunia, dyschezia and infertility. As such, endometriosis not only has a significant impact on the lives of millions of women and their families, but also is associated with an enormous socioeconomic burden [15, 19].

It is generally accepted that endometriosis presents in three different entities, which are frequently found together: peritoneal lesions, deep endometriosis and ovarian endometriotic cysts (endometriomas) [14]. Endometriomas are probably the most commonly diagnosed form of endometriosis because of the relative ease and accuracy of ultrasound diagnosis. Although their exact prevalence and incidence are not known, they have been reported in 17–44% of women with endometriosis [4]. The presence of ovarian endometriomas has been reported as being a marker for deep endometriosis [16] and multifocal deep vaginal, intestinal and ureteric lesions [5].

The pathogenesis of endometriomas remains contentious, with a variety of theories proffered, including invagination and subsequent collection of menstrual debris from endometriotic implants, which are located on the ovarian surface and adherent peritoneum [2, 10], colonisation of functional ovarian cysts by endometriotic cells [12] and coelomic metaplasia of the invaginated epithelial inclusions [14].

Endometriomas frequently present a clinical dilemma as to whether and how to treat them when found during imaging. Overall, currently available treatment options for all types of endometriosis include oestrogen suppression, progestins, surgery or a combination of these [8]. Surgical treatment is the mainstay of endometrioma management when treatment is required, aimed at the elimination of endometriotic tissue, to provide sufficient tissue for histological assessment and to preserve a maximum amount of normal ovarian tissue (where fertility is desired and/or risk of menopause is to be avoided). It has been shown that surgical treatment of endometriotic cysts is associated with the unintentional removal or destruction of ovarian follicles, which can be objectified by a measurable post-operative reduction in serum anti-Müllerian hormone (AMH) levels or antral follicle count (AFC) on ultrasound [1, 20].

## Materials and methods

Previously published guidelines have provided recommendations on the management of endometriosis, based on the best available evidence [7, 11, 22]. However, these guidelines were not intended to provide recommendations on the technical details of surgical procedures. Therefore, the European Society for Gynaecological Endoscopy (ESGE), the European Society of Human Reproduction and Embryology (ESHRE) and the World Endometriosis Society (WES) have formed a working group to provide a series of recommendations on the practical aspects of the different surgical procedures for the treatment of endometriosis.

This document is the first in a series of recommendations covering technical aspects of different methods of surgery for different entities of endometriosis and will focus on endometriomas in women of reproductive age; recommendations dealing with other forms of endometriosis will be addressed in separate subsequent publications. These recommendations should be read in conjunction with the aforementioned evidence-based guidelines on the clinical management of endometriosis.

Owing to the scarcity of evidence, these recommendations are mostly based on expert opinion on best clinical practice. The techniques described here may have different levels of efficacy in achieving individualised management goals; hence, background factors such as the woman’s age, her symptoms (pain, infertility), primary aim of the treatment (eliminating/improving pain, improving fertility, ruling out malignancy), ovarian reserve, unilaterality/bilaterality, number and size(s) of the cyst(s), associated conditions (deep endometriosis, uterine or ovarian abnormalities and in infertile couples: tubal status and male factor) and history of previous surgery (i.e. recurrence) will need to be taken into consideration when a decision for surgery is made and the type of technique is chosen.

In addition to the recommendations, the working group has set up a web platform with videos on the different options available for surgery of ovarian endometrioma. The web platform is accessible through the following link (https://www.eshre.eu/surendo) or via the ESGE, ESHRE and WES websites.

## Recommendations

### Anatomical considerations

Endometriomas are frequently stuck densely to surrounding structures such as the ipsilateral pelvic side wall, the fallopian tube, the posterolateral uterus and the bowel. As part of preoperative planning, the surgeon should consider the possibility of hydro-ureters and asymptomatic hydronephrosis. The ureter enters the small pelvis by crossing the iliac vessels and then courses anteriorly in the peritoneum of the pelvic side wall directly under the ovary. Ovaries with endometriotic cysts are usually adherent to the ovarian fossa, where the ureter may also be involved in the disease. Occasionally, ureteric obstruction can be seen at this point. This will need to be taken into account during surgery.

The ovary receives its blood supply from two sources: the ovarian artery, which arises from the abdominal aorta below the renal artery and laterally approaches the ovary through the suspensory ovarian (infundibulopelvic) ligament, and an anastomosis between the ovarian artery and the ascending branch of the uterine artery/tubal artery in the ovarian ligament. Thus, the larger intra-ovarian vessels are found in the anterolateral aspect of the ovary—the hilum at the insertion of the mesovarium. The surgeon needs to be aware of this and, in particular for endometrioma involving that area, has to possess the skills to avoid excessive bleeding which might lead to destruction of healthy ovarian tissue through cauterisation and disruption of ovarian blood supply.

### General recommendations

- Assess the possible extent of disease and the size, number and location (unilateral or bilateral) of the ovarian endometriotic cysts before surgery. Meticulous preoperative planning is part of the procedure and should include:
  - A bimanual examination to check adnexal masses and endometriotic nodules
  - Pelvic ultrasound (and/or MRI) to determine
    - The number, size and location (unilateral or bilateral) of the cysts
    - Presence of endometriotic nodules
    - Extent of the Pouch of Douglas obliteration
    - Presence of hydronephrosis
    - Presence of hydrosalpinx
  - Ovarian reserve tests (AFC, AMH) when future fertility is a concern
- Assess serum tumour markers in case of suspicion of malignancy at imaging, as this may be helpful to exclude malignancy. The risk of unexpected malignancy is small but may need to be taken into consideration. The accuracy of serum tumour markers may be limited as some of these are raised in the presence of endometriosis [13].
- Obtain appropriate consent from the woman before surgery. She should be fully informed of all possible risks associated with the surgical procedure, including general risks of laparoscopic surgery, a potentially reduced ovarian reserve, and the (albeit small) risk of loss of the ovary and consequences thereof. Although still controversial, the woman should also be informed about the possibility of preoperative freezing of oocytes, especially in the case of bilateral disease [21].
- Refer the woman to a centre of expertise where the necessary surgical expertise is available, if the surgery cannot be performed or completed safely [11].
- Handle the ovarian tissue as atraumatically as possible.
- Be aware of the risk of damage to ovarian reserve in endometrioma surgery. Fertility preservation should be considered when the reserve is already compromised.
- Consider using anti-adhesion measures such as oxidised regenerated cellulose, polytetrafluoroethylene surgical membrane and hyaluronic acid products, as these may be beneficial in reducing postoperative adhesion formation [7, 22]. Ovarian suspension could be an alternative method of adhesion prevention [9].

### Initial stages of laparoscopic surgery for ovarian endometriomas

- Inspect the pelvic organs, upper abdomen and appendix.
- Obtain peritoneal washings and biopsies before mobilising the ovary with endometrioma in the presence of clinically relevant ascites, suspicious peritoneal lesions or ovarian cysts of abnormal appearance. However, for a presumed endometrioma, peritoneal washing is not routinely recommended.
- Consider using three laparoscopic working ports as these may facilitate surgery.
- Separate the ovary with endometrioma from the pelvic side wall, where it is usually adherent to, by adhesiolysis. This usually results in drainage of the endometrioma. It is important to visualise the ureter at this stage to avoid damage, as the ovary may be adherent to it. In the presence of dense adherence, start the surgery by dissecting the ureter from the healthy tissue proximal to the adherence point. Endometriotic tissue on the pelvic side wall will need to be removed as well (this will be covered in the subsequent recommendation on the treatment of peritoneal endometriosis).
- Where the cyst ruptures, extend the opening in the cyst wall adequately to expose the cyst cavity. Multiple incisions and excessive opening should be avoided to prevent damaging the ovarian cortex, functional ovarian tissue and the hilum. Where feasible, the cyst may be turned inside out to facilitate further treatment.
- When the ovary is not adherent, the incision should ideally be over the thinnest part of the ovarian endometriotic surface or, if this is not visible, on the antimesenteric border.
- Irrigate and inspect the cyst cavity to rule out malignancy. Any suspicious area should be biopsied for histological confirmation of any diagnosis.
- If suspicious for malignancy, local guidelines for further management should be followed.
- Irrigate and aspirate thoroughly to check for haemostasis and to remove any remaining cyst fluid or blood clots from the abdominal-pelvic cavity.

The following options are available for conservative surgical treatment of ovarian endometrioma:

- Cystectomy
- Ablation by laser or by plasma energy
- Electrocoagulation

These methods, the combined technique and the two- or three-step approach are described below.

### Cystectomy

- After mobilisation of the ovary and drainage of the cyst, make an incision to reveal the cleavage plane; this may be either on the edge of the cyst opening or a central incision, which divides the cyst into two halves. With both approaches, the incision should be away from the blood vessels in the hilum/meso-ovarium. Use of cold cut at the edge of the cyst opening may assist in identifying the cleavage plane.
- To aid dissection and identification of the cyst wall, saline or diluted synthetic vasopressin solution (0.1–1 unit/ml) may be injected under the cyst capsule. The diluted synthetic vasopressin injection has the additional advantage of reduced bleeding during cyst removal. Synthetic vasopressin is not available in all countries and, although rare, may cause intraoperative cardiovascular complications including bradycardia and hypertension.
- In some cases, a cleavage plane may not easily be identified after the ovarian incision. In such cases, it may be better to take a small part of the cyst wall for histological diagnosis and then use an ablation method, rather than risking damage to the ovary from persistent attempts to perform cystectomy.
- Once the cleavage plane is identified, use gentle traction and counter-traction with appropriate instruments to dissect the cyst capsule from the ovarian parenchyma. Traction and counter-traction may be effective during the initial part of the dissection. Avoid use of excessive force to separate a highly adherent cyst from the ovary, as this will likely cause tearing of the ovarian tissue, excessive bleeding, plus the need for coagulation or diathermy, and thus further damage the normal ovarian tissue.
- Careful identification of the cleavage plane and precise spot bipolar coagulation is the key to achieve haemostasis, to prevent unnecessary damage to healthy tissue and to avoid blind or excessive diathermy.
- Ensure final haemostasis after complete removal of the cyst capsule. Bipolar coagulation, suturing or intraovarian haemostatic sealant agents may also be used for this purpose. It is important to avoid damaging the major blood supply at the hilum coming in from the ovarian and infundibulopelvic ligaments at this stage.
- After removal of large endometriomas, it may be necessary to reconstruct the ovary and achieve haemostasis with monofilament sutures. For small endometriomas, suturing is often not required as the ovarian opening usually approximates spontaneously. If a suture is used, it should ideally be placed inside the ovary, as the exposed suture may be prone to adhesion formation.
- Small cyst walls may be divided and retrieved directly through a port. Large cyst walls can be removed in a specimen retrieval bag. Posterior colpotomy is very rarely used for retrieval of endometriomas.

### Laser ablation

- Ablate the entire inner surface of the cyst wall using the laser beam. Power settings of 30–55 W for CO_2_ laser beam and 6–10 W for CO_2_ fibre (based on animal data) are usually used. The laser should be on the ablate function to widen the beam (e.g. ‘defocus’ or ‘surgiscan’). The laser should be applied in such a mode that it can ablate the tissue while preserving the underlying healthy tissue.
- Aim to vaporise the endometriotic cyst lining only until haemosiderin pigment-stained tissue is no longer visible (until the colour changes from reddish to yellow-white). The entire depth of the cyst capsule does not need vaporisation, as endometriotic tissue is present only superficially.
- Use intermittent irrigation to maintain good visibility and to remove carbon debris.
- Ensure the border of the cyst opening is completely vaporised.

### Plasma energy ablation

- Ablate the entire inner surface of the cyst wall using plasma energy in coagulation mode set at 10 to 40, at a distance averaging 5 mm from the tip of the hand piece [17, 18].
- Aim to vaporise the endometriotic cyst lining only until haemosiderin pigment-stained tissue is no longer visible (until the colour changes from reddish to yellow-white). The entire depth of the cyst capsule does not need vaporisation, as endometriotic tissue is present only superficially.
- Take care to treat all areas and to ablate the edges of the invagination site.
- When cyst eversion is not feasible, expose the cyst interior progressively to apply the plasma at an angle perpendicular to the inner surface of the cyst.

### Electrocoagulation

Electrosurgery is widely used for the treatment of ovarian endometrioma. Coagulation modes with different techniques and electrodes lead to different voltage levels, including modulation of high-frequency (HF) current with soft coagulation, forced coagulation or spray coagulation. These various application modes result in different effects on the target tissue and cause different degrees of tissue damage (Table 1).

- Coagulate the cyst lining systematically using bipolar forceps. The power setting depends on the generator and type of forceps used, but a 25–40 W setting is frequently used. It is advisable to start at a lower power setting and adjust it depending on the effectiveness of coagulation achieved. The key point is to use very short coagulation times to minimise ovarian tissue damage, as the depth of the destruction can be difficult to judge.
- Monopolar energy may be used in selected areas where there is fibrotic endometriotic tissue located at the hilum. A power setting of 15–20 W is frequently used.
- Tissue damage tends to be deeper than with laser and plasma energy ablation; hence, the ovary should be cooled frequently with irrigation fluid.

**Table 1:** Principles of electrosurgery for endometrioma

**Electrosurgery application**
The thickness of the capsule of an endometrioma can be up to 3.0 mm and varies between cysts, but may also change within the same cyst. During the application of HF energy for destruction of an endometriotic lesion by a thermal effect, it is difficult to assess the changes in the tissue. Whereas the impact on superficial tissue may be visible by change of colour and vaporisation, coagulation of deeper structures is more difficult to observe. The surgeon needs to be aware of the exact HF effect of each instrument and various application forms. Coagulation or vaporisation of the ovarian cyst should inactivate endometriotic lesions superficially and respect the underlying tissue. Uncontrolled application of heat or deep coagulation may result in destruction of healthy tissue, primordial follicles and/or blood supply of the ovary, with severe consequences for ovarian function.
**Monopolar energy**
*Cutting current* is unmodulated alternating current, and vaporises or cuts the tissue for superficial ablation and deeper coagulation effect. *Coagulation current* is modulated alternating high voltage current and has a higher thermal spread, which leads to deeper coagulation of the tissue. *Blended current* is a mixture of cutting and coagulation currents, and is generated by altering the time that the current is applied. The more concentrated the energy, the greater is the thermodynamic effect. Current density depends on the size of the electrode (a smaller electrode may require a lower power setting). Use of monopolar diathermy, with a low power setting and small contact surface, provides better control of the tissue effect.
**Argon beam coagulation**
With this instrument, ionised argon gas carries electrons from the electrode to the tissue. The gas stream produces a monopolar tissue effect depending on the diameter of the beam and the distance between the beam and the target. The tissue effect is similar to that achieved by monopolar coagulation, but allows treatment of wider superficial areas.
**Bipolar energy**
Bipolar diathermy is a very useful technique to coagulate endometriosis in a safer way than monopolar diathermy. The current passes across the tissue between the two jaws of the instrument. The tissue temperature could be up to 300 400°C at the point of maximum current flow. The penetration into the tissue can be up to 10 12 mm, depending on the power setting and the application time.

### Combined technique

A combined technique using both excision and ablation can be used to prevent excessive bleeding and ovarian tissue removal/damage from the ovarian hilum, particularly for larger endometriomas.

- Open and drain the cyst followed by identification of the cleavage plane, as described above.
- Strip 80–90% of the cyst wall and perform a partial cystectomy, as described above, up to the ovarian hilum. Laser, plasma energy or bipolar can then be applied to treat the remaining endometriotic tissue (10–20%).
- Suturing of the ovary may be considered to restore anatomy.

### Two- or three-step approach for large endometriomas

For large endometriomas, a two- or three-step procedure can be considered.

- The first step involves opening and draining the endometrioma as described in the initial stage section.
- Inspect the cyst cavity and take a biopsy.
- Following this initial step, administer a GnRH agonist (GnRHa) therapy for 3 months, during which time the thickness of the cyst wall significantly decreases, with atrophy and reduction in stromal vascularisation of the cyst [6].
- Complete the surgery with a second laparoscopy in the form of either cystectomy, CO_2_ vaporisation, bipolar diathermy or plasma ablation of the cyst wall lining.

Although women have to undergo two invasive procedures, the potential benefit is that this may facilitate the management of larger ovarian endometriomas, reduce recurrence rates and limit decrease in ovarian reserve.

### Further considerations

Laparotomy is rarely indicated for benign ovarian endometriomas, regardless of the diameter of the cyst and/or the associated adhesions [11]. If the procedure is too difficult to perform by laparoscopy, it is better to stop the procedure after the drainage of the endometrioma(s), prescribe GnRHa for 3 months, and re-operate 3–6 months later. Alternatively, the woman may be referred to a centre with the necessary surgical expertise [11].

Oophorectomy may be considered after careful discussion with the woman, particularly in the presence of recurrent or large unilateral endometriomas, or suspicion of potential malignancy. Informed consent, as described above, needs to be obtained in all cases, and fertility concerns need to be discussed.
